# Author Correction: Vocal state change through laryngeal development

**DOI:** 10.1038/s41467-020-14760-9

**Published:** 2020-02-24

**Authors:** Yisi S. Zhang, Daniel Y. Takahashi, Diana A. Liao, Asif A. Ghazanfar, Coen P. H. Elemans

**Affiliations:** 1 Princeton Neuroscience Institute, Princeton University, Princeton, NJ 08544 USA; 2 Department of Psychology, Princeton University, Princeton, NJ 08544 USA; 3 Department of Ecology & Evolutionary Biology, Princeton University, Princeton, NJ 08544 USA; 4 Department of Biology, University of Southern Denmark, 5230 Odense M, Denmark

**Keywords:** Development of the nervous system, Neurophysiology, Biomechanics

Correction to: *Nature Communications* 10.1038/s41467-019-12588-6, published online 9 October 2019.

In the original version of the Article, three Supplementary Movies were inadvertently omitted. The main text also referred to them as “Supplementary Movie S1” rather than “Supplementary Movie 1”, etc. This has now been corrected in the PDF and HTML versions of the Article.

