# Supplementary material for: Vocal state change through laryngeal development
Source: Nat Commun. 2019 Oct 9;10:4592. doi: 10.1038/s41467-019-12588-6 (PMC6785551; doi:10.1038/s41467-019-12588-6)
Supplement: Supplementary file 1 — Supplementary Information [file 41467_2019_12588_MOESM1_ESM.pdf]

Supplementary Information

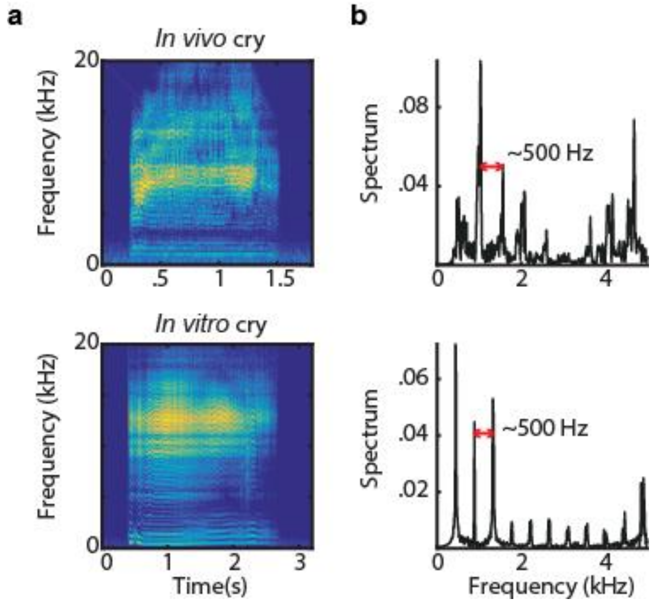

Supplementary Figure 1 | Comparison between *in vivo* cry and *in vitro* cry. a, Spectrograms of a marmoset infant cry (top) and cry-like sound generated from unstretched infant larynx (bottom). **b,** Spectra of the *in vivo* cry (top) and *in vitro* cry (bottom). Both sounds are featured by a harmonic stack with ~500 Hz f_0.

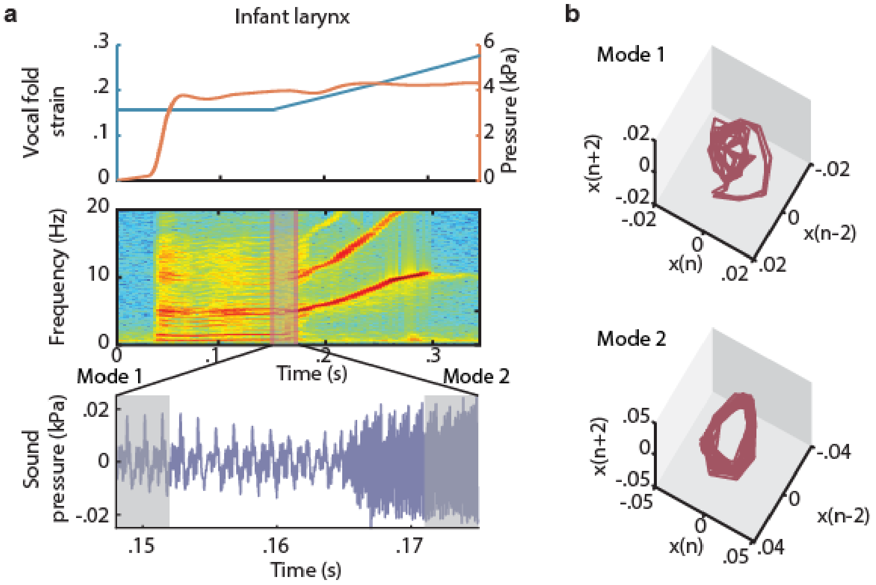

Supplementary Figure 2 | Two distinct vibratory modes during increasing vocal fold length. a, Zoom-in view of acoustic signal in Figure 2D around the transition from low strain to high strain. **b,** Phase portraits of the two vibratory modes.
