# Supplementary material for: Vocal state change through laryngeal development
Source: Nat Commun. 2019 Oct 9;10:4592. doi: 10.1038/s41467-019-12588-6 (PMC6785551; doi:10.1038/s41467-019-12588-6)
Supplement: Supplementary file 3 — Reporting Summary [file 41467_2019_12588_MOESM3_ESM.pdf]

natureresearch

Corresponding author(s): *Double-blind peer review submissions: write DBPR and your manuscript number here instead of author names.*

Last updated by author(s): *YYYY-MM-DD*

Reporting Summary

Nature Research wishes to improve the reproducibility of the work that we publish. This form provides structure for consistency and transparency in reporting. For further information on Nature Research policies, see Authors & Referees and the Editorial Policy Checklist .

Statistics

For all statistical analyses, confirm that the following items are present in the figure legend, table legend, main text, or Methods section.

n/a	Confirmed	
☐	☒	The exact sample size (n) for each experimental group/condition, given as a discrete number and unit of measurement
☐	☒	A statement on whether measurements were taken from distinct samples or whether the same sample was measured repeatedly
☐	☒	The statistical test(s) used AND whether they are one- or two-sided<br>*Only common tests should be described solely by name; describe more complex techniques in the Methods section.*
☐	☒	A description of all covariates tested
☐	☒	A description of any assumptions or corrections, such as tests of normality and adjustment for multiple comparisons
☐	☒	A full description of the statistical parameters including central tendency (e.g. means) or other basic estimates (e.g. regression coefficient) AND variation (e.g. standard deviation) or associated estimates of uncertainty (e.g. confidence intervals)
☐	☒	For null hypothesis testing, the test statistic (e.g. F, t, r) with confidence intervals, effect sizes, degrees of freedom and P value noted<br>*Give P values as exact values whenever suitable.*
☒	☐	For Bayesian analysis, information on the choice of priors and Markov chain Monte Carlo settings
☒	☐	For hierarchical and complex designs, identification of the appropriate level for tests and full reporting of outcomes
☒	☐	Estimates of effect sizes (e.g. Cohen's d, Pearson's r), indicating how they were calculated

Our web collection on statistics for biologists contains articles on many of the points above.

Software and code

Policy information about availability of computer code

Data collection	MATLAB R2016a, Motionstudio v2.10.01, PlexControl
Data analysis	MATLAB R2018a, ilastik 1.3.0

For manuscripts utilizing custom algorithms or software that are central to the research but not yet described in published literature, software must be made available to editors/reviewers. We strongly encourage code deposition in a community repository (e.g. GitHub). See the Nature Research guidelines for submitting code & software for further information.

Data

Policy information about availability of data

All manuscripts must include a data availability statement. This statement should provide the following information, where applicable:

- Accession codes, unique identifiers, or web links for publicly available datasets
- A list of figures that have associated raw data
- A description of any restrictions on data availability

The datasets that support the findings of the current study will be available in the DRYAD Digital repository.

Field-specific reporting

Please select the one below that is the best fit for your research. If you are not sure, read the appropriate sections before making your selection.

☒ Life sciences ☐ Behavioural & social sciences ☐ Ecological, evolutionary & environmental sciences

For a reference copy of the document with all sections, see nature.com/documents/nr-reporting-summary-flat.pdf

Life sciences study design

All studies must disclose on these points even when the disclosure is negative.

Sample size	Our sample size of n=8 was chosen based on previous studies on excised primate larynx experiments, e.g. Brown, Charles H., et al. "Laryngeal biomechanics and vocal communication in the squirrel monkey (Saimiri boliviensis)." The Journal of the Acoustical Society of America 113.4 (2003): 2114-2126. No sample size calculation was performed.
Data exclusions	n=2 excised larynges (not included in the 8) were excluded because no sound was obtained.
Replication	The described effect was observed in every single sample we tested. No further replication attempt was performed.
Randomization	Samples were allocated into groups by age. Samples from both sexes and different families were used.
Blinding	There was no blinding procedure. The servo motor parameters were the same for each age group, but different for different age groups.

Reporting for specific materials, systems and methods

We require information from authors about some types of materials, experimental systems and methods used in many studies. Here, indicate whether each material, system or method listed is relevant to your study. If you are not sure if a list item applies to your research, read the appropriate section before selecting a response.

Materials & experimental systems

n/a	Involved in the study	
☒	☐	Antibodies
☒	☐	Eukaryotic cell lines
☒	☐	Palaeontology
☐	☒	Animals and other organisms
☒	☐	Human research participants
☒	☐	Clinical data

Methods

n/a	Involved in the study	
☒	☐	ChIP-seq
☒	☐	Flow cytometry
☒	☐	MRI-based neuroimaging

Animals and other organisms

Policy information about studies involving animals; ARRIVE guidelines recommended for reporting animal research

Laboratory animals	One 6-year-old female common marmoset (Callithrix jacchus), and specimens from 4 adult (>2 yrs), 3 infant (newborns) and 1 juvenile (7 mos) deceased marmosets.
Wild animals	The study did not involve wild animals.
Field-collected samples	The study did not involve samples collected from the field.
Ethics oversight	All experiments were approved by the Princeton University Institutional Animal Care and Use Committee (Protocol #1908-18). We obtained licenses to import (License nr: 2016-12-719-05377) and work with animal tissue (License nr: DK-08-123-oth-107) from the Danish Ministry of Food.

Note that full information on the approval of the study protocol must also be provided in the manuscript.
