# Supplementary material for: Vocal state change through laryngeal development
Source: Nat Commun. 2019 Oct 9;10:4592. doi: 10.1038/s41467-019-12588-6 (PMC6785551; doi:10.1038/s41467-019-12588-6)
Supplement: Supplementary file 4 — Description of Additional Supplementary Files [file 41467_2019_12588_MOESM4_ESM.docx]

**Description of Additional Supplementary Files**

File Name: Supplementary Movie 1

Description: High-speed films of an infant larynx oscillating at the low-strain region. The area bounded by the cranial edge of the vocal fold is labeled in red and the area bounded by the caudal edge is labeled in blue. The frame rate was reduced by 2000 times (originally was at 30 kHz).

File Name: Supplementary Movie 2

Description: High-speed films of an infant larynx oscillating at the high-strain region. The area bounded by the cranial edge of the vocal fold is labeled in red and the area bounded by the caudal edge is labeled in blue. The frame rate was reduced by 1000 times (originally was at 30 kHz).

File Name: Supplementary Movie 3

Description: High-speed films of an adult larynx oscillating at the low-strain region. The area bounded by the cranial edge of the vocal fold is labeled in red and the area bounded by the caudal edge is labeled in blue. The frame rate was reduced by 1000 times (originally was at 30 kHz).
